# 25C-NBOMe Ingestion

**DOI:** 10.5811/cpcem.2017.5.33994

**Published:** 2017-10-03

**Authors:** Jonathan Zygowiec, Spencer Solomon, Anthony Jaworski, Michael Bloome, Ari Gotlib

**Affiliations:** Henry Ford Wyandotte Hospital, Department of Emergency Medicine, Wyandotte, Michigan

## Abstract

The popularity of recreational synthetic drug use has increased within the past several years. Emergency physicians, along with prehospital providers, are often the first to interact with patients who use these new drugs. We report the case of a 27-year-old male with two emergency department visits with confirmed ingestion of a relatively new synthetic drug of abuse. We discuss symptom management as well as the identification process of the ingestant.

## INTRODUCTION

Within the past several years the popularity of recreational synthetic drugs has risen significantly, leading to an increase of toxicological emergencies in the United States.^1^ People typically gain access to these drugs via the Internet, at gas stations or convenience stores.^1^ Emergent testing for these compounds proves difficult as these agents are not yet widely known by healthcare professionals and no rapid laboratory tests exist to identify them. These drugs can be ingested via tablet, capsule, sprays, eye droppers or as blotter papers that the user places under his tongue. Their street names include “legal acid,” “Mr. Happy” and “smiley paper.” Many of these drugs are consumed by younger individuals attending parties or music festivals*.*^2^

One of the newer classes of synthetic drugs are 2C agents, which are a modification of the hallucinogens known as phenethylamines. One of the compounds belonging to the 2C class that has become a synthetic drug of abuse is 2-(4-iodo-2,5-dimethoxyphenyl)-N- [(2-methoxyphenyl) methyl] ethanamine (also known as either 25I-NBOMe or “N-Bomb”).^3^ Through their very potent serotonin 2A receptor (5-HT2A) agonist activity,^4^ 2C agents are known for their hallucinogenic and stimulant effects. Chemical manipulation of the parent structure can result in more potent effects.^5^ Since the discovery of this class, many such derivatives have been detected. With the higher potency of NBOMe derivatives compared to the 2C parent compound, certain adverse events associated with overdose (i.e., psychomotor agitation, altered mental status, rhabdomyolysis, and seizures) are more frequent.^1^

The differences in potency among the known derivatives and appropriate treatments are currently being studied. To date, a handful of case reports exist describing 25C-NBOMe.^2^,^6^–^8^ We describe two cases of intoxication with NBOMe (one of which was confirmed as 25C) in the same patient where high dose benzodiazepines were used to control the patient’s symptoms and prevented major adverse reactions from the synthetic drug.

## CASE REPORT

A 27-year-old man was brought to the emergency department (ED) in police custody for medical evaluation. The police officers reported that they were called to a gas station after the patient was observed acting aggressively and confrontationally with patrons. He was observed attempting to enter several vehicles in the parking lot. When police arrived, the patient resisted arrest and was physically restrained.

Upon evaluation, the patient was alert and aggressive, requiring police officers and the hospital security guards to physically restrain him. His initial vital signs were blood pressure 139/90 mm Hg, heart rate 146 beats per minute (bpm), respiratory rate 28 breaths/min, temperature 36.6ºC, peripheral capillary oxygen saturation 98% on room air. There was no evidence of trauma, his mucous membranes and skin were dry, and he was tachycardic and agitated. An electrocardiogram was obtained, blood was drawn, urine was obtained via catheterization, and an intravenous (IV) fluid bolus of two liters of 0.9% normal saline was given.

When contacted, the Michigan Poison Control Center recommended IV fluid hydration, cardiopulmonary monitoring, and benzodiazepines as necessary. He was given 2 mg lorazepam and 5 mg of haloperidol lactate in the ED. As the patient’s mentation improved, he admitted to using “N-Bomb.” The remainder of the ED encounter was unremarkable and the patient was admitted to the hospital for continuous monitoring and psychiatry evaluation. It was noted that on the medical floor the next day the patient had removed his IV and telemetry leads and could not be located.

The same patient returned to the ED via ambulance approximately three weeks later after he was found wandering the streets. The patient admitted to ingestion of 2–4 tablets of “25I-NBOMe,” four tablets of lisdexamfetamine, and an unknown amount of fluoxetine. Upon presentation the patient was very agitated and pulled his monitor cords off. The patient’s vital signs were temperature 36.8^0^ C, heart rate 120 bpm, blood pressure 153/119 mm Hg, and respiratory rate 22 breaths/min. On exam his pupils were approximately 4 mm and reactive bilaterally; he did not display myoclonus or sweating. He was given lorazepam 4 mg IV push for symptom control and given 1 L bolus of 0.9% normal saline. Laboratory studies were drawn, including a send-out qualitative serum assay for NBOMe. Poison control was contacted and recommended increasing doses of benzodiazepines for symptomatic control.

During the patient’s ED stay, he continued to be very agitated and combative with staff while constantly attempting to leave the ED and remove his IV catheter. As the patient started to hallucinate, he requested supplies to draw pictures. The patient continued to be symptomatic and eventually required lorazepam 16 mg IVP, after which he became calm. Despite the large doses of lorazepam, the patient’s vital signs remained stable and he did not require airway support. He was admitted to the hospital for continued monitoring and psychiatric evaluation. Over a 4.5-hour timeframe, the patient received a total of 34 mg of lorazepam (doses of 4 mg, 2 mg, 8 mg, and 16 mg) with improvement in symptoms.

The following morning, the patient’s primary care physician evaluated him, but he was lethargic and unable to provide any history. The patient was fully alert on the second day of admission, and psychiatry was able to evaluate him and recommended transferring him to an inpatient substance abuse center. The patient refused and signed out against medical advice approximately 48 hours after he presented to the ED. Approximately a week after leaving, the qualitative liquid chromatography result came back positive for 25C-NBOMe, resulting above the 0.50 ng/mL reporting limit. The lab rest requires a lavender top tube, which undergoes high-performance liquid chromatography-tandem mass spectrometry; the results take approximately three days and the cost listed is $165. (NMS Labs, Willow Grove, PA (www.nmbslabs.com).

## DISCUSSION

In early 2010, the synthetic drugs known as “N-Bombs” were widely available for recreational use.^9^ These compounds, with their high affinity for 5-HT_2A_ receptors, have potent hallucinogenic properties. The 25C derivative of NBOMe was first reported by Ettrup et al. in 2011, and continued reports of 25C-NBOMe ingestion described patients with neuropsychiatric and autonomic symptoms, including tachycardia, tachypnea, dilated pupils, diaphoresis and hyperthermia. A case of a fatal 25C overdose was reported in 2015.^7^ Nevertheless, difficulties in the recognition of the NBOMe toxicology persist due to lack of familiarity with the agents and limited access to emergent confirmatory lab testing.

**CPC-EM Capsule**

What do we already know about this clinical entity?

A relatively new synthetic drug, NBOMe, has potentially lethal outcomes even at small doses. There is no rapid testing to identify the substance.

What makes this presentation of disease reportable?

Emergency physicians are often the first clinicians to interact with these new synthetic drugs. Understanding the presentation, acute management, and testing options is important.

What is the major learning point?

Ingestions can require large doses of benzodiazepines to control symptoms. They can often present as a co-ingestion. Identification of the substance is by a send-out lab and takes several days.

How might this improve emergency medicine practice?

Emergency medicine is a dynamic specialty. Being aware of new synthetic drugs and the limitations of identification and symptom control is important.

Confirmatory lab testing by the method of high-performance liquid chromatography-tandem mass spectrometry is available but few laboratories have the ability to test for these compounds. This qualitative blood test can detect the presence of the 25I, C, H, and B derivatives of NBOMe. Drug levels above 0.5ng/mL are considered positive results. In this case, the patient self-reported taking 25I-NBOMe as well as the other medications. Rapid testing for these agents was not available at our hospital at the time of treatment in the ED and the patient had to be treated symptomatically. His blood was transported to an affiliated tertiary center for a complete toxicology screen. The serum results came back days after the patient’s initial encounter, and it was noted that the patient tested negative for 25I-NBOMe but was positive for the 25C derivative.

Previous cases describe treatment strategies that include IV fluids and benzodiazepines, with the resolution of symptoms typically ranging between 10 hours to three days after presentation.^2^,^8^ In this case, with the immediate and aggressive treatment of the patient with escalating doses of benzodiazepines, the symptoms of agitation were treated and controlled. Additionally, the patient did not develop hyperthermia, rhabdomyolysis, seizures, or any other complications of 25C-NBOMe overdose.

## CONCLUSION

In conclusion, this case report supports that the toxidrome associated with 2C derivatives are similar to sympathomimetic and serotonergic toxidromes, and aggressive treatment with benzodiazepines can be effective. Testing for these new synthetic drugs can be difficult to obtain and do not directly affect management or treatment, although obtaining confirmatory testing can lead to awareness of what is being used locally.
